# Annual cycle of mesozooplankton at the coastal waters of Cyprus (Eastern Levantine basin)

**DOI:** 10.1093/plankt/fbac075

**Published:** 2023-01-20

**Authors:** G Fyttis, S Zervoudaki, A Sakavara, S Sfenthourakis

**Affiliations:** Department of Biological Sciences, University of Cyprus, Aglantzia 2109, 20537, Nicosia, Cyprus; Institute of Oceanography, Hellenic Centre for Marine Research (HCMR), 46,7 km Athens Sounio ave., 19013 Anavyssos, Attiki, Greece; Institute of Oceanography, Hellenic Centre for Marine Research (HCMR), 46,7 km Athens Sounio ave., 19013 Anavyssos, Attiki, Greece; Department of Marine Sciences, University of the Aegean, 81100 Mytilene, Lesvos island, Greece; Department of Biological Sciences, University of Cyprus, Aglantzia 2109, 20537, Nicosia, Cyprus

**Keywords:** mesozooplankton, phenology, cyprus, levantine, Eastern Mediterranean

## Abstract

This study is the first to explore monthly and seasonal succession of the zooplankton community in coastal waters of Cyprus using a 12-month period time series. A total of 192 taxa of mesozooplankton (MZ), 145 of which were copepods, were identified at three sites at the southern and one site at the northern coasts of the island. Zooplankton distribution and community structure were influenced mostly by stratification, temperature and Chl-a. The combination of upwelling and advection from the Rhodes Gyre during summer, causing cooler waters in the southern coast of Cyprus, seems to control the food supply and offered favorable feeding conditions to zooplankton, enhancing their numbers. The proximity to a fish farm also positively affected MZ abundance and biomass. This study also revealed the importance of smaller species (e.g. *Clausocalanus paululus*) and juvenile stages (e.g. *Clausocalanus*, *Oithona* and *Corycaeus* spp.) in composition, structure and functionality of the copepod community. These species seems to be more important in low Chl-a environments, where the relative size of primary consumers is expected to be smaller and the microbial components dominant. This baseline study paves the way for further investigation of the elements of marine food webs in the ultra-oligotrophic environment of the Eastern Mediterranean.

## INTRODUCTION

Zooplankton play an essential role in controlling ocean production and biogeochemistry (Banse, 1995) and occupy a key position in the pelagic food web by transferring organic carbon produced by unicellular algae through photosynthesis to higher levels such as the pelagic fish stocks (Lenz, 2000). Seasonal zooplankton dynamics and the driving mechanisms of their variability are, at present, a central issue of oceanographic research. The identification of changes in species composition related to long-term trends in the ocean is a strategy used to monitor the influence of global changes on marine communities (Fernández de Puelles et al., 2003). Hence, due to the ecological importance of zooplankton in marine ecosystems, the study of the spatial and seasonal variability of coastal zooplankton communities, and their relation with environmental conditions, is important in order to improve our understanding of the function of coastal marine ecosystems (Terbiyik and Polat, 2013; Zervoudaki et al., 2007).

The Mediterranean Sea is an enclosed basin, where evaporation exceeds precipitation (Bergamasco and Malanotte-Rizoli, 2010) and characterized by strong temperature and salinity gradients between the western Mediterranean (WMED) and the eastern Mediterranean (EMED) (Fedele et al., 2022), caused by its complex multiscale circulation (Robinson et al., 2001). A west–east gradient in nutrient deficiency that also exists between the two basins (Powley et al., 2017), with the eastern Mediterranean characterized as a “marine desert” based on its very low Chlorophyll-a (Chl-a) concentrations (Azov, 1991), and its easternmost basin, the Levantine, being ultra-oligotrophic (Pitta et al., 2005). The ultra-oligotrophic nature of the Levantine basin is reflected in the low suspended POC concentrations (Thingstad et al., 2005a) and extremely low primary production rates (Psarra et al., 2005). The average phytoplankton productivity is approximately half of the productivity of other oligotrophic areas of the world’s oceans (Béthoux, 1989; Krom et al., 2003). Oligotrophy seems to result from the very low concentration of inorganic phosphorus, which is assumed to limit primary production (Thingstad et al., 2005b). Hence, a west-to-east decrease of standing stock of zooplankton has been reported (Dolan et al., 2002; Siokou-Frangou, 2004).

Cyprus, located in the Eastern Levantine basin is characterized by high seawater salinity and temperature (Krom et al., 2014), some of the globally lowest nutrient (Petrou et al., 2012) and Chl-a concentrations (Bianchi et al., 1996) in surface layers and coastal waters, respectively. The ultra-oligotrophic characteristics of the Levantine basin are further reinforced in Cyprus by the extremely limited runoffs in its coastal waters, due to the frequent prolonged periods of drought (Xevgenos et al., 2021) and the high number of dams (108 in total) constructed along the majority of streams of the island (Water Development Department, 2022). In the coastal waters of Cyprus, filaments of cooler water are seen to extend offshore from Cape Akrotiri mainly during the summer period. This is the result of upwelling and advection from the Rhodes Gyre caused by the off-shore transport of water during the summertime by strong, steady northwesterly winds (Zodiatis et al., 2008). These changes in coastal shallow water mass movements and the aforementioned extent of land influences (e.g. rivers and sewage flow) may affect the dynamics of plankton communities (Terbiyik and Polat, 2013).

Several studies on the seasonal variability of zooplankton have been conducted in different coastal regions of the Mediterranean Sea (Calbet et al., 2001; Mazzocchi et al., 2011; Yebra et al., 2022; Zervoudaki et al., 2020). However, little information and few data are available regarding zooplankton in Cyprus (Siokou-Frangou et al., 1997; Hannides et al., 2015a; Hannides et al., 2015b; Samuel-Rhoads et al., 2016; Vasilopoulou et al., 2022). Despite the available information on zooplankton in Cyprus open waters, there is still a lack of information regarding monthly variability of mesozooplankton communities and detailed species composition in the coastal waters.

This study aims to provide information on the mesozooplankton (MZ) annual cycle, through temporal resolution of sampling (on a monthly basis) and to correlate these findings with environmental variables. This study constitutes also one of the first attempts to record MZ species and abundance in coastal waters of Cyprus aiming to offer a baseline for future long-term monitoring of marine food webs, and to contribute to the Marine Strategy Framework Directive (MSFD).

## METHOD

### Sampling area

**Fig. 1:**
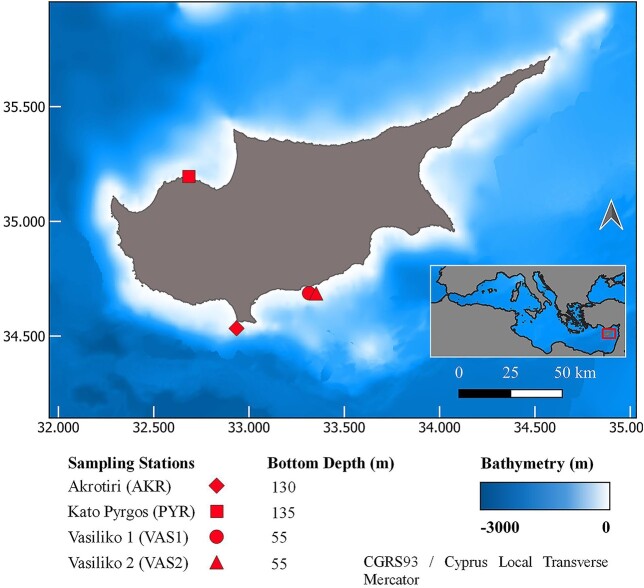
Map of sampling stations.

Monthly sampling was carried out in three coastal sites of Cyprus (Akrotiri, Kato Pyrgos and Vasiliko, Fig. 1) for 12 consecutive months (January—December 2016). These areas were selected on the basis of their differences in temperature (southern vs northern coasts) and anthropogenic pressures (Vasiliko). Overall, four sampling stations were set (Fig. 1). The area of Vasiliko is one of the most impacted coastal areas in Cyprus. Among the industries located there, are a cement production factory, an abandoned chemical fertilizers industry, a power station, the largest desalination plant of the island and the majority of aquacultures (fish-farms) in Cyprus (Moksness et al., 2013). Hence, in order to check the impact of anthropogenic pressures on MZ communities, a sampling station (VAS1) was selected near a fish-farm. Another station (VAS2), located ~3.5 km from the aquaculture, was also selected for comparing the structure of MZ communities with VAS1. Station Akrotiri (AKR) is located off the Akrotiri peninsula in the southern coasts of Cyprus, where annual strong upwelling is being reported during the summer period (Mauri et al., 2019), whereas, station Kato Pyrgos (PYR) is located on the northwest of Cyprus. The distance from the shore of the four sampling stations are 3 km for AKR, 1.2 km for PYR, 3 km for VAS1 and 4 km for VAS2.

### Sampling collection and analysis

Vertical profiles of temperature and salinity, were carried out with continuously measurements from the surface down to the bottom in each sampling station using a Sea Bird Electronics SBE 19 plus CTD. A 5 L Niskin bottle was used to collect seawater at discrete depths (2, 10, 20, 50, 75, 100 m) to estimate the concentration of nutrients and Chl-a in the water column. Immediately after sampling, water for Chl-a analysis from each sampling depth is filtered onto GF/F filters (0.45 μm, 47 mm) and stored at −20°C until further analysis. Analysis of nitrate (NO_3_^−^), nitrite (NO_2_^−^), ammonium (NH_4_^+^) and phosphate (PO_4_^3−^) was carried out according to Strickland and Parsons (1972) on an OI Analytical Flow Solution IV+ autoanalyzer and Chl-a analyzed with High Performance Liquid Chromatography (HPLC) analysis.

The sampling of MZ was carried out using a WP2 closing net (0.255m^2^ mouth diameter, 200 μm mesh size) by carrying out two vertical tows, 0–50 m and 50–100 m in AKR and PYR, and one (0–50 m) in VAS1 and VAS2. A flowmeter was used to estimate the volume of the filtered water. Sampling was carried out at the same time (approximately 7.30 am) every month, ensuring daylight, to avoid community differences due to zooplankton vertical migration. Samples were fixed immediately after collection and preserved in a 4% final formaldehyde–seawater solution, buffered with borax sodium tetraborate (Harris et al., 2000). Each sample was divided in the laboratory using a Motoda Box Splitter into two sub-samples for the estimation of MZ biomass (dry weight, mg m^−3^) and abundance (ind m^−3^), as well as for species identification. The subsample for bulk biomass measurement was filtered onto pre–weighed and pre-combusted, glass–fiber filters (Whatman GF/C) and dried at 60°C for 24 h (Postel et al., 2000). The dry weight (mg) of samples was calculated from the difference between the final weight and the weight of the filter. The biomass (mg DW m^−3^) was extrapolated from the total volume sampled by the net. A stereoscope (Olympus SZX16) with brightfield, variable oblique illumination and darkfield with zoom ratio 16.4:1, zoom range 7–115 (1x objective, 10x eyepiece and 20x eyepiece that were applied, when necessary, in order to achieve greater magnification e.g. zoom up to 230) and a microscope (ZEISS Primo Star) were used for the identification of MZ to the lowest possible taxonomic level. Copepods were identified at a species level, whenever possible while other groups were mainly identified at higher taxonomic levels. Aliquots of samples analyzed, ranged between 10 and 20%, depending on the sample density.

### Data analysis

All statistical analysis and data manipulations were performed with Past 4.09 (Hammer et al., 2001) and R programming language (R Core Team, 2021). Each season was defined as the average of the three successive months traditionally associated with the four seasons in oceanography, i.e. January–February–March for winter, April–May–June for spring, July–August–September for summer and October–November–December for autumn (Levitus, 1982). The vertical profiles of total temperature, salinity, PO_4_^−3^, DIN, N:P and Chl-a for all stations were created with the interpolation of existing data in specific depths and months. For this analysis, the packages “raster” (Hijmans, 2022) and “plot3D” (Soetaert, 2021) were used. One-way ANOVA was performed with PAST 4.09 software for scientific data analysis, to test differences in abiotic and biotic parameters among stations, seasons and between the surface (0–50 m) and the deeper layer (50–100 m), followed by a post-hoc Tukey’s test (α = 0.05) to compare individual groups. Data were checked for normal distribution (Shapiro–Wilk test, α = 0.05) and homogeneity of variance (Levene’s test, α = 0.05). Data were log-transformed or a square root transformation was applied when needed before statistical analysis. The non-parametric test of Kruskal–Wallis (α = 0.05) was used when the one-way ANOVA assumptions of normality and homogeneity were not met. The Shannon diversity index (H′, Shannon, 1948) was calculated along with species richness of copepods (S, defined as number of species). The non-parametric test of Spearman’s rank correlation was used to check correlations among abiotic and biotic elements, as the assumption of **normal distribution,** was not met. The package “Hmisc” was used for this analysis. Principal component analysis (PCA) was carried out based on the environmental parameters (temperature, salinity, PO4^−3^, DIN, N:P and Chl-a) in order to identify the principal environmental factors, controlling similarities between layers (0–50 m and 50–100 m) and among seasons at layer 0–50 m. For the creation of the biplots the PAST software was used. In order to identify underlying ecological gradients in the MZ communities in the marine layer 0–50 m, the non-metric Multi-Dimensional Scaling (nMDS) was applied. To perform this analysis the function “metaMDS” was used. The ordination analysis was followed by a permanova test, an analysis of variance that is used for partitioning distance matrices among potential sources of variation. This analysis was performed to test if the taxa composition had statistically significant differences among sampling seasons, by using the function “adonis2.” To better understand how the MZ communities vary between seasons a multilevel pairwise comparison (~permanova) was conducted for each pair of sampling seasons with the use of the function “pairwise.adonis.” Moreover, the stations’ ordination scores were fitted as independent variables into, three continuous variables, namely the temperature, the salinity and the Chl-a concentration and three factor variables namely stations’ location, sampling season and sampling month, as dependent variables. Each variable was tested separately, with the function “envfit.” Only vectors with significant correlation are shown (*P* < 0.05) and the centroids of the stations based on sampling season. In all methods mentioned above, that require a distance matrix as an input, the Bray–Curtis dissimilarity matrix was chosen, because it takes into account both species presence/absence and their abundance. Hellinger’s ecological transformation was applied to the abundance data prior to those analysis, with the function “decostand” (Legendre and Gallagher, 2001).

The statistical significance in all the above methods was tested by using 999 permutations and all methods mentioned above were performed with the package “vegan” (Oksanen et al., 2022) in R, except pairwise.adonis for which “pairwise Adonis” package was used (Arbizu, 2017). In order to identify indicator taxa among the four sampling seasons and locations the function “multipatt” that is found in the package “indispecies” was used. This function calculates an indicator value index for each taxon that is the product of two components, called “A” and “B.” The component “A” represents the positive predictive value of a taxon as indicator for a group and the component “B” represents the probability of finding that taxon in stations belonging to that group (De Cáceres and Legendre, 2009; Dufrêne and Legendre, 1997). By default, this analysis is conducted for each species independently, and combinations of taxa are not taken into account. Moreover, rare taxa in a site belonging to a certain group will not be identified as indicator taxa from this analysis.

In order to evaluate the effects of environmental drivers on the most dominant taxa (abundance data) a generalized linear model was applied using the mvabund package in R (Wang et al., 2012; Wang et al., 2021). The explanatory variables included in the full GLM model were salinity, temperature, chlorophyll, NP, DIN, season (4-level factor, winter, spring, summer and autumn), station (4-level factor, AKR, VAS1, VAS2 and PYR) and depth (2-level factor, 50 and 100 m). To remove multicollinearity, the Spearman rank coefficient (*r* > 0.7) was calculated, and one variable (PO_4_) was excluded due to its correlation with NP (*r* = − 0.767, *P* < 0.01). Model selection was carried out using the “drop1” function with the removal of non-significant terms, until the final model comprised only statistically significant variables (significance level set at α = 0.05). Univariate tests were then performed to determine which dominant taxa showed a significant effect in response to the environmental drivers. *Isias clavipes* was excluded from the analysis due to extreme overdispersion on its abundance data.

The same procedure was followed for zooplankton biomass, abundance, taxa and MZ groups (Copepoda, Pteropoda, Ostracoda and Chaetognatha—these groups met the assumption of normality) using the MASS package (Venables and Ripley, 2002). Negative binomial distribution was chosen for all models, except taxa count data, for which poison was used. In addition, comparisons between null and final models by ANOVA confirmed the goodness of fit of the best models. The normality of residuals and response plots were checked to ensure the GLMs did not violate model assumptions (Zuur et al., 2010). Prior to model building, data exploration indicated missing values that were excluded from our dataset.

## RESULTS

### Spatial and temporal distribution of environmental parameters

**Fig. 2:**
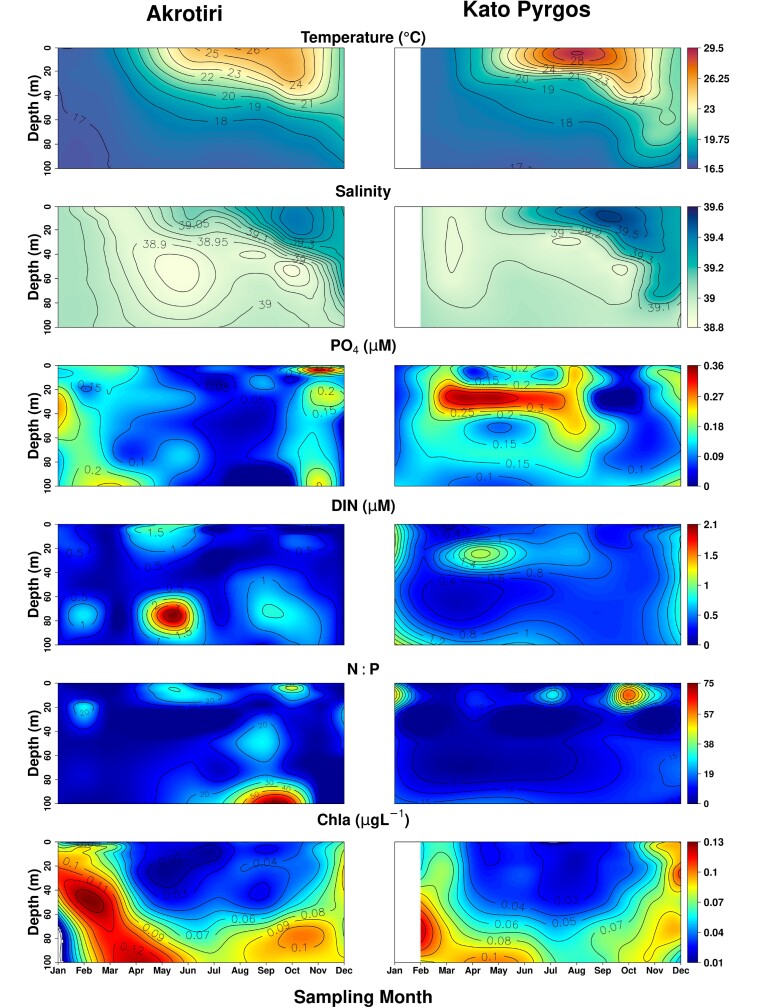
Vertical profiles of total temperature, salinity, PO_4_, DIN, N:P and Chl-a, in Akrotiri (AKR) and Kato Pyrgos (PYR).

**Fig. 3:**
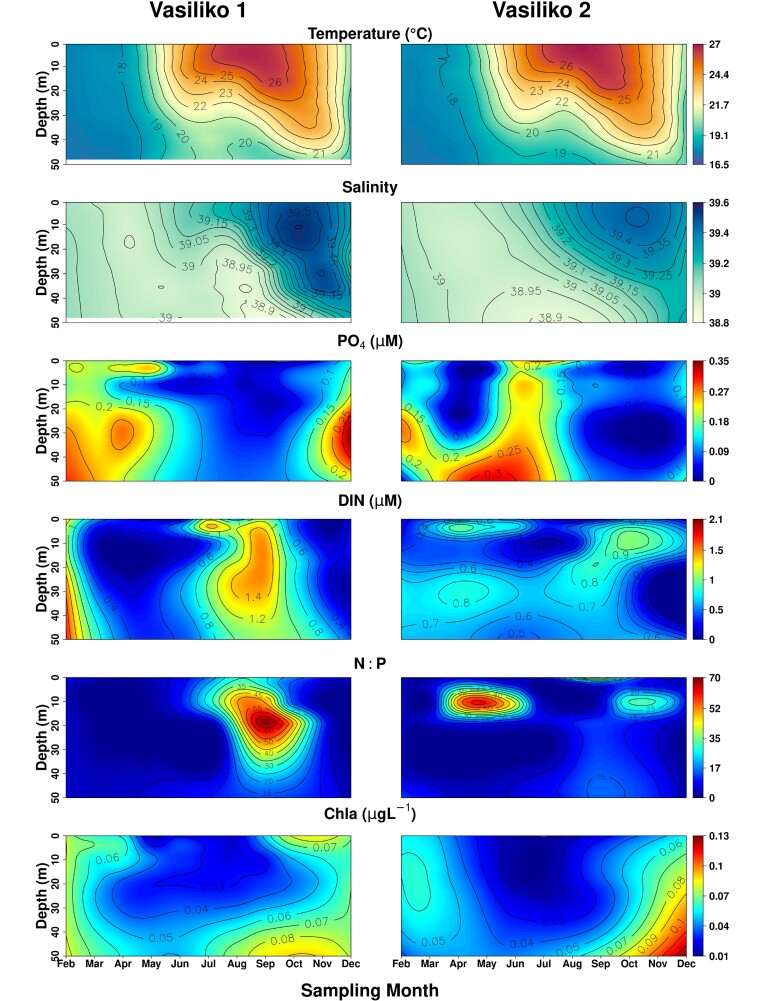
Vertical profiles of temperature, salinity, PO_4_, DIN, N:P and Chl-a, in Vasiliko 1 (VAS1) and Vasiliko 2 (VAS2).

The monthly variation of temperature, salinity, nutrients (PO_4_^−3^, DIN, N:P) and Chl-a are presented in Fig. 2 for AKR and PYR, and in Fig. 3 for VAS1 and VAS2. The analysis of *in situ* data showed an evident seasonal pattern in temperature; temperatures surpassed 29°C during summer at the surface waters of PYR, and dropped to 17°C during winter at a depth of 100 m in AKR. The temperature profiles depicted the mixed water-column overall, lasting from late-autumn until early spring, while a strong stratification was observed for the rest of the year, with a marked seasonal thermocline between 20 and 50 m depth at AKR and PYR and between 10 and 40 m at VAS1 and VAS2. Salinity was high throughout the year (>38.7), as expected for the high salinity Levantine waters. Temperature displayed a statistically significant positive correlation with salinity (*rs =* 0.52, *P* < 0.001) and N:P (*rs =* 0.51, *P* < 0.001), and a negative correlation with PO_4_^−3^ (*rs = −*0.39, *P* < 0.01) and Chl-a (*rs = −*0.49, *P* < 0.001) (Table I). The minimum concentration of PO_4_^−3^ was 0.007 μM for all stations, whereas the maximum was 0.316 μM in autumn at AKR (Fig. 2). DIN ranged from 0.02 to 3.841 μM overall. Concentrations of DIN and PO_4_^−3^ did not vary significantly with depth, or among seasons and stations. No significant correlation was found between DIN and salinity or temperature. The mean N:P ratio for all stations was 6.86 in winter, 11.74 in spring, 14.03 in summer and 13.59 in autumn. Chl-a ranged between 0.005 and 1.4 μgL^−1^ overall, with a clear increasing gradient proportional to increasing depth. The maximum values of Chl-a were recorded during winter at 75 and 100 m depth, at AKR and PYR. Lower values of Chl-a were observed during summer in the upper layer overall.

**Table I:** Spearman’s correlation coefficients (*rs*) among the abiotic and biotic elements found in this study. Only statistically significant values are shown with ^*^

	Temperature	Salinity	PO_4_^3−^	N:P	Chl-a	Total Abundance
Temperature		0.52^*^^*^^*^	-0.39^*^^*^	0.51^*^^*^^*^	-0.49^*^^*^^*^	
						
Total abundance			-0.3^*^			
Biomass						0.6^*^^*^^*^
Diversity					0.38^*^^*^	0.45^*^^*^^*^
Appendicularia					0.27^*^	0.42^*^^*^
Chaetognatha	-0.30^*^	-0.38^*^^*^				0.39^*^^*^
Cladocera	0.83^*^^*^	0.38^*^^*^				0.28^*^
Cnidaria						0.68^*^^*^^*^
Copepoda						0.92^*^^*^
Copepoda males						0.75^*^^*^^*^
Copepoda juv.	0.43^*^^*^					0.83^*^^*^^*^
Polychaeta						
Pteropoda	0.45^*^^*^					

^*^: *P* < 0.05; ^*^^*^: *P* < 0.01; ^*^^*^^*^: *P* < 0.001.

### Mesozooplankton abundance and biomass

**Fig. 4:**
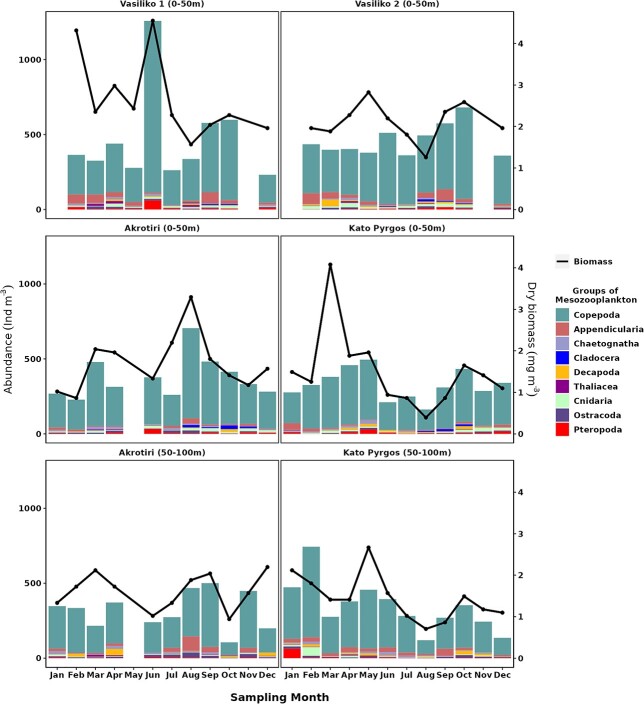
Annual pattern of total abundance (ind m^−3^) and biomass of zooplankton (mgDW m^−3^) (lines) and dominant zooplankton groups (stacked bars) at 0–50 m layer in Vasiliko 1 (VAS1) and Vasiliko 2 (VAS2), Akrotiri (AKR) and Kato Pyrgos (PYR) stations and at 50–100 m layer in Akrotiri (AKR) and Kato Pyrgos (PYR).

Figure 4 shows the abundance (individuals m^−3^) and biomass as dry weight (mg m^−3^) variation of the main MZ groups in the layer 0–50 m for all stations and in the layer 50–100 m at the deeper stations. Total MZ abundance ranged between 106–1257 ind. m^−3^ with an average of 382 ± 171.68 ind. m^−3^ overall. Fluctuations of total abundance were not consistent among stations, seasons or depth (e.g. a clear peak of abundance observed in spring at VAS1 constituted the highest among all the stations, Table SI), which resulted mostly from the increase of *Isias clavipes*. No significant differences were found among the stations or seasons, regarding abundance. Total abundance exhibited a positive correlation with biomass (*rs =* 0.6, *P* < 0.001) (Table I). The highest biomass occurred in spring in VAS1, following the trend of total abundance (Τable SII). Significant differences (*P* < 0.001) of biomass were displayed between stations at 0–50 m layer, whereas differences in the deep layers were found only in summer.

### Composition of MZ community

A total of 192 taxa of MZ were found overall in this study, and 145 copepod taxa were identified among them. (Table SIII). Copepoda constituted by far the most abundant group ranking between 67.7–92.6%, with an average of 81.3 ± 5.8%, overall, followed by Appendicularia (5.5 ± 4.7%, ranking between 0.21–20.1%) and Cnidaria (2.8 ± 1.8%, ranking between 0.6–8.1%). Minor groups such as Pteropoda, Decapoda (larvae), Ostracoda, Chaetognatha and Cladocera, constituted 8.6% of total MZ abundance (Fig. 4). Copepoda presented higher abundance in spring, but did not exhibit any other clear seasonal pattern among the stations. Some groups, like Cladocera and Appendicularia, showed seasonality; the former were present mainly in summer and autumn, and the latter occurred in higher abundances in winter and summer. Moreover, Pteropoda and Chaetognatha were more abundant in spring (Table VI). Α clear vertical pattern was observed only for Appendicularia and Ostracoda in AKR and PYR.

**Fig. 5:**
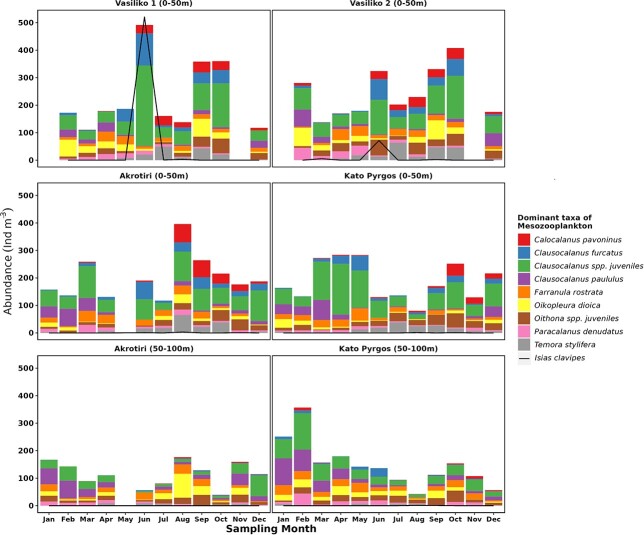
Annual variation of the abundance (ind m^−3^) of dominant taxa overall throughout the year.

The dominant taxa of the MZ community were *Clausocalanus* spp. juveniles, *Clausocalanus paululus*, *Oithona* spp. juveniles, *Farranula rostrata*, *Oikopleura dioica*, *Clausocalanus furcatus*, *Temora stylifera*, *Calocalanus pavoninus*, *Paracalanus denudatus* and *Isias clavipes* (Fig. 5).

Αmong the 145 taxa of Copepoda that were identified in this study, a total of 120 were found in AKR, 122 in PYR, 97 in VAS1 and 91 in VAS2 (Table SΙV). The Shannon–Wiener (H′) diversity index was similar among stations and varied between 2.2 and 3.7, whereas no seasonal or bathymetric or spatial pattern was observed (Table SV).

The copepod community constituted overall by two main orders, Calanoida (68.5%) and Cyclopoida (31.1%), displaying 84 and 52 taxa, respectively. The other three minor groups: Harpacticoida, Monstrilloida, Siphonostomatoida, had minor contribution to the total copepod abundance (Table SVI). Calanoida showed their higher abundances in VAS1 and VAS2 in spring and autumn, respectively, whereas they presented higher abundances in summer at AKR and lower at PYR, demonstrating a different pattern.

Calanoida were composed mainly of *Clausocalanus* spp. juveniles (16.3 ± 8.4%), *C. paululus* (5.3 ± 6.1%), *C. furcatus* (4.2 ± 3.9%), *Temora stylifera* (3.1 ± 4.5%) and *Calocalanus pavoninus* (2.7 ± 3.5%) (Table SΙII). *Clausocalanus* spp. juveniles exhibited high abundance, in all seasons except summer, when their lowest abundance was recorded, mostly in AKR and PYR. *Clausocalalanus* species (*C. paululus, C. parapergens C. arcuicornis, C. jobei, C. lividus* and *C. mastigophorus*) showed a decreasing seasonal pattern from winter to autumn. However, *C. furcatus* presented a different seasonal trend presenting higher abundances in late spring, summer and autumn. *T. stylifera* exhibited a clear seasonal pattern, as its abundances started to increase in late spring until summer. *C. pavoninus* showed higher abundances in summer and autumn. Regarding the vertical distribution, all the above species showed higher abundance in the shallow layer, whereas *Calocalanus styliremis*, *Haloptilus longicornis* and *Mesocalanus tenuicornis* showed an increasing trend in the deeper layer in comparison with the upper one.

Cyclopoida showed a seasonal increasing trend from winter to autumn in VAS1 and VAS2, whereas they presented higher abundances in summer at AKR and lower at PYR, the same trend as Calanoida, regarding this season. They presented an increasing trend at the layer 50–100 m, in winter, spring and summer, in both stations AKR and PYR. The Cyclopoida were represented mostly by *Oithona* spp. juveniles (5.3 ± 3.2%), *Farranula rostrata* (4.7 ± 2.6%), *Corycaeus* spp. juveniles (2.4 ± 1.7%), *Oithona plumifera,* (1.4 ± 2.4%), *Oncaea scottodicarloi* (1.4 ± 1.5%) (Table SIII). *Oithona* spp. juveniles exhibited an increasing seasonal trend through the year. *Farranula rostrata* presented its highest abundances in spring, whereas *Agetus flaccus*, *A. limbatus* and *A. typicus*, were higher in winter and spring. *O. plumifera* is found mainly in autumn, whereas *Oithona longispina* exhibited higher abundances in spring and summer. *O. scottodicarloi* showed higher abundance in spring. Regarding the vertical distribution, *Oithona* spp. juveniles had higher abundances in the deeper layer of AKR almost throughout of the year and in PYR during winter and spring. *Corycaeus* spp. juveniles, *Sapphirina metallina* and some species of Oncaeidae (*O. scottodicarloi*, *O. mediterranea*, *Oncaea* spp. males, *Oncaea* spp. juveniles, *O. venusta, Triconia conifera, T. hawii*) presented higher abundances in the deeper layer in comparison with upper layer in AKR and PYR. Some of the Copepoda that were found in the present study are characterized as alien species according to Zenetos et al. (2006), Zenetos et al. (2012) and Zenetos and Galanidi (2020). These are *Calanopia elliptica*, *Centropages furcatus*, *Euchaeta concinna*, *Triconia rufa* and *Triconia hawii.* The first four species were found once or twice while the sampling period with few specimens recorded, whereas the latter, *Triconia hawii* presented higher abundances overall and mainly in summer and autumn. *Triconia rufa* was found in winter, *Euchaeta concinna* in spring and *Calanopia elliptica* and *Centropages furcatus* in autumn.

### Relationships between MZ and environmental variables

**Fig. 6:**
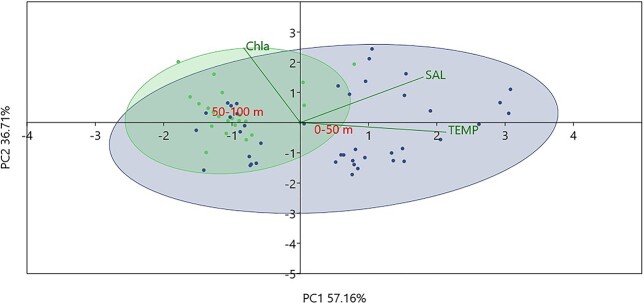
PCA based on the environmental parameters (TEMP: temperature, SAL: salinity, Chla: Chl-a, between layers 0–50 m and 50–100 m overall).

**Fig. 7:**
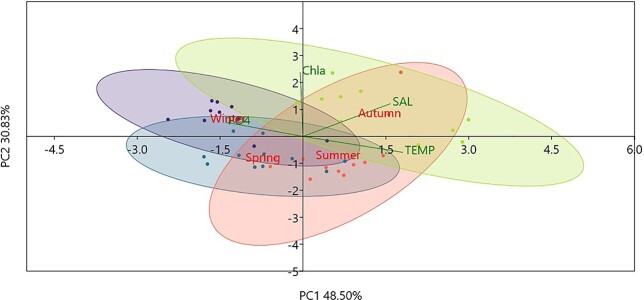
PCA based on the environmental parameters (TEMP: temperature, SAL: salinity, Chla: Chl-a, PO4: PO_4_^3−^ among seasons: winter, spring, summer and autumn, on the upper layer (0–50 m) overall.

PCA values in Fig. 6 showed that the first two axes explain 93.3% of the variance. The bathymetric layer 0–50 m is driven mainly by temperature and salinity, whereas the layer 50–100 is influenced by Chl-a. PCA in Fig. 7 showed that the first two axes explain 79.3% of the variance. PO_4_^−3^ mainly affected the stations in winter, temperature and salinity the stations in summer and autumn.

A strong positive correlation among abiotic and biotic parameters was displayed between temperature and Cladocera (*rs =* 0.83, *P* < 0.05) (Table I).

**Table II:** MZ taxa that characterize the different sampling seasons and their combination, with the “A” and “B” components, the values of indicator value index (stat) and their significance, as revealed from “indispecies” analysis.

	**A**	**B**	**stat**	***P* value**	**sig**
**Winter**					
*Pleuromamma gracilis*	0.6823	0.8000	0.739	0.0006	^*^^*^^*^
*Haloptilus* ssp. *juveniles*	1.0000	0.4000	0.632	0.0048	^*^^*^
*Paracalanus* ssp. *juveniles*	0.6585	0.6000	0.629	0.0082	^*^^*^
*Lucicutia gemina*	0.7871	0.5000	0.627	0.0027	^*^^*^
*Candacia simplex*	0.8107	0.4000	0.569	0.0077	^*^^*^
*Calocalanus contractus*	0.8183	0.3000	0.495	0.0287	^*^
**Spring**					
*Onychocorycaeus latus*	0.8726	0.4545	0.63	0.0043	^*^^*^
**Autumn**					
*Clio* spp.	1.0	0.6	0.775	0.0003	^*^^*^^*^
*Ischnocalanus.gracilis*	1.0	0.3	0.548	0.0195	^*^
**Autumn + Spring + Summer**					
*Temora stylifera*	0.9312	1.0000	0.965	0.0001	^*^^*^^*^
*Oithona tenuis*	0.9171	0.8788	0.898	0.0040	^*^^*^
*Acartia negligens*	0.9436	0.8485	0.895	0.0011	^*^^*^
*Calocalanus pavonicus*	0.9354	0.8485	0.891	0.0045	^*^^*^
*Limacinidae*	0.9223	0.8485	0.885	0.0042	^*^^*^
*Evadne spinifera*	1.0000	0.7273	0.853	0.0001	^*^^*^^*^
*Onychocorycaeus ovalis*	0.9662	0.5758	0.746	0.0127	^*^

^*^: *P* < 0.05; ^*^^*^: *P* < 0.01; ^*^^*^^*^: *P* < 0.001.

**Fig. 8:**
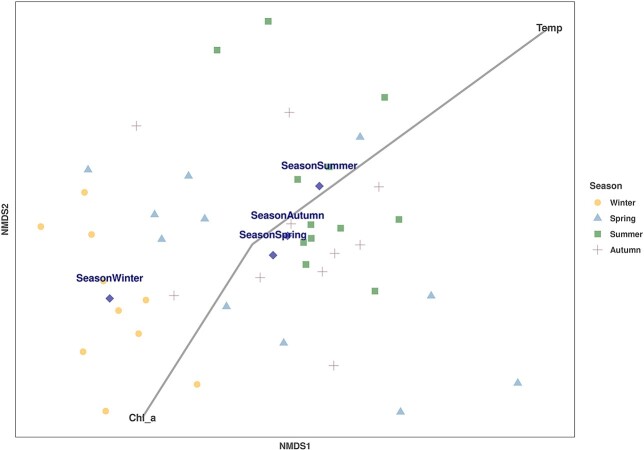
NMDS analysis of MZ communities on the upper water column layer (0–50 m) overall (stress value ≈ 0.16). The stations are color and shape coded according to sampling season. The centroid values for all stations belonging to a certain season are plotted as well. The lines represent regression vectors for environmental variables with correlation to the MZ community composition. Line length represents correlation strength, and the line angle shows the direction of sample’s increase with respect to that variable.

NMDS analysis of the MZ communities on the upper water column layer (0–50 m) revealed a separation of stations sampled in winter from the other with three seasons (Fig. 8). The Goodness of fit in combination with the Permanova test showed that there is a weak, but statistically significant correlation between MZ communities and sampling season (envifit *r*^2^ ~ 0.4, *P* = 0.001 and Permanova, *P* = 0.001) (Table SVII). Moreover, taxa composition presented significant correlation with both temperature and Chl-a (envifit, *r*^2^ = 0.76—*P* = 0.001, envifit, *r*^2^ = 0.19—*P* = 0.017, respectively). A strong correlation was observed with temperature and a weak with Chl-a.

The “indispecies” analysis (Table II) revealed that *Pleuromamma gracilis, Haloptilus* ssp. juveniles, *Paracalanus* ssp. juveniles, *Lucicutia gemina*, *Candacia simplex* and *Calocalanus contractus* are strongly and significantly associated with winter, the taxon *Onychocorycaeus latus* with spring, whereas the taxa *Clio* spp. and *Ischnocalanus.gracilis* were found to be good indicators for stations sampled in autumn. None of the taxa that were identified in this study found to be indicator for summer. In addition, taxa such as *Temora stylifera*, *Oithona tenuis, Acartia negligens, Calocalanus pavonicus, Limacinidae, Evadne spinifera* and *Onychocorycaeus ovalis,* exhibited abundance pattern more associated to a combination of seasons spring, summer and autumn. A higher number of taxa act as indicators for winter in comparison with the other three seasons, which have common indicator species. The “indispecies” analysis showed no specific spatial pattern regarding the distribution of MZ taxa, as only two taxa found to be weak indicators for different stations, *Macrosetella gracilis* for AKR (A: 1.0000, B: 0.2727, stat:0.522, p = 0.047) and *Calocalanus contractus* for VAS1 (A: 0.7899, B: 0.3000, stat: 0.487, p = 0.047). *Clausocalanus mastigophorus* was found to be an indicator for stations located in the South (AKR, VAS1 and VAS2) (A: 0.8766, B: 0.7742, stat: 0.824, p = 0.025).

The GML analysis on the MZ community data of the most dominant taxa revealed four environmental variables, as the most important parameters significantly correlated with the distribution of MZ (Table III). In particular, depth correlated significantly to the distribution of the four dominant taxa (*C. pavoninus*, *C. furcatus*, *Clausocalanus* spp. juveniles and *T. stylifera*), whereas season was the factor that was significantly correlated to the distribution patterns of five dominant taxa (*C. pavoninus, C. paululus*, *Oithona* spp. juveniles, *P. denudatus* and *T. stylifera*). In addition, temperature presented significant correlation with the distribution of five dominant taxa (*C. pavoninus*, *C. furcatus*, *C. paululus, Clausocalanus ssp.* juveniles and *T. stylifera*), whereas Chl-a correlated significantly to the distribution only of one dominant species, *C. paululus*. *Oikopleura dioica* and *Faranula rostrata* did not seem to be affected by the four environmental parameters (Table IV).

**Table III:** Analysis of deviance table resulting from the GLM analysis of the nine dominant species abundance data in relation to the environmental variables. Residual degrees of freedom: Resid. Df, degrees of freedom: Df, deviance: Dev and *P*-value: Pr(>Dev).

Explanatory variables	Resid. Df	Df	Dev	Pr(>Dev)
Depth	54	1	74.29	0.001^*^^*^^*^
Season	51	3	169.74	0.001^*^^*^^*^
Temperature	50	1	93.04	0.001^*^^*^^*^
Chlorophyll	49	1	29.15	0.014^*^

^*^: *P* < 0.05; ^*^^*^: *P* < 0.01; ^*^^*^^*^: *P* < 0.001.

**Table IV:** Results from GLM analysis of the nine dominant species abundance data associated with the explanatory variables. Deviance: Dev and *P*-value: Pr(>Dev). All *P*-values < 0.05 are in bold.

Explanatory variables		Depth	Season	Temperature	Chl-a
Dominant taxa					
*C. pavoninus*	Dev	13.651	11.265	27.411	0.126
	Pr(>Dev)	**0.006**	**0.094**	**0.001**	0.977
*C. furcatus*	Dev	28.411	8.501	18.696	0.54
	Pr(>Dev)	**0.001**	0.14	**0.001**	0.947
*C. paululus*	Dev	0.856	37.083	10.717	16.8
	Pr(>Dev)	0.859	**0.002**	**0.017**	**0.003**
*Clausocalanus* ssp. juveniles	Dev	12.18	9.878	17.099	4.762
	Pr(>Dev)	**0.007**	0.135	**0.001**	0.254
*F. rostrata*	Dev	0.292	13.079	0.611	0.054
	Pr(>Dev)	0.859	0.053	0.821	0.977
*Oikopleura dioica*	Dev	0.237	5.976	0.003	5.286
	Pr(>Dev)	0.859	0.209	0.991	0.216
*Oithona* spp. juveniles	Dev	0.979	14.813	3.068	0.066
	Pr(>Dev)	0.859	**0.034**	0.31	0.977
*P. denudatus*	Dev	0.94	26.821	0.022	0.571
	Pr(>Dev)	0.859	**0.003**	0.991	0.947
*T. stylifera*	Dev	16.738	42.321	15.411	0.942
	Pr(>Dev)	**0.003**	**0.001**	**0.001**	0.927

In addition, GLM results showed that MZ biomass was associated with the factor of stations and in particular with VAS1 and VAS2 at which biomass was greater. Total MZ abundance was positively affected by warmer temperatures and sampling season (winter and spring) which showed higher MZ abundance. On the other hand, MZ taxa were found to be negatively affected by warmer temperatures and positively affected by the concentration of Chl-a. The number of MZ taxa was also associated with the stations and in particular with VAS2 (Table V). Copepoda were associated with the season and specifically with winter and spring, whereas Pteropoda with spring and mostly by temperature. Ostracoda were negatively associated with salinity, and the seasons spring and winter, where presented low abundance. Finally, Chaetognatha were positively correlated to factor season and in particular to spring, where they demonstrated higher abundance (Table VI).

**Table V:** Summary results of the best GLMs for MZ biomass, abundance (negative binomial distribution was used to account for data overdispersion) and taxa (Poisson distribution was used for count data) associated with explanatory variables.

Dependent variable	Explanatory variables	Estimate	SE	*z*	Explained deviance (D^2^)	AIC	*P* value (ANOVA null vs final)
Biomass	Intercept	5.02	0.10	49.28^*^^*^^*^	45.96	620.93	^*^^*^^*^
	StationVAS1	0.51	0.13	3.89^*^^*^^*^			
	Station VAS2	0.30	0.13	2.26^*^			
Abundance	Intercept	3.84	0.53	7.25^*^^*^^*^	26.27	714.89	^*^^*^^*^
	Temperature	0.09	0.02	3.77^*^^*^^*^			
	Season spring	0.44	0.15	2.90^*^^*^			
	Season winter	0.47	0.17	2.84^*^^*^			
Taxa	Intercept	4.21	0.17	25.09^*^^*^^*^	32.49	398.6	^*^^*^^*^
	Temperature	-0.02	0.01	-2.10^*^			
Chla	2.20	0.69	3.17^*^^*^				
Station VAS2	0.15	0.05	2.81^*^^*^				

^*^: *P* < 0.05; ^*^^*^: *P* < 0.01; ^*^^*^^*^: *P* < 0.001. For ANOVA, null vs final model *P* value <0.05 indicates that the final model is better than the null model.

**Table VI:** Summary results of the best GLMs for MZ groups (negative binomial distribution was used to account for data overdispersion) associated with explanatory variables

Dependent variable	Explanatory variables	Estimate	SE	*z*	Explained deviance (*D*^2^)	AIC	*P* value (ANOVA null vs final)
Copepoda	Intercept	3.21	0.65	4.94^*^^*^^*^	16.81	618.31	^*^^*^^*^
	Season spring	0.48	0.18	2.61^*^^*^			
	Season winter	0.53	0.21	2.57^*^			
	Temperature	0.07	0.03	2.33^*^			
Pteropoda	Intercept	-3.38	1.18	-2.85^*^^*^	44.51	308.88	^*^^*^^*^
	Season spring	1.01	0.30	3.34^*^^*^^*^			
	Temperature	0.24	0.05	4.40^*^^*^^*^			
	DIN	0.68	0.27	2.48^*^			
	NP	-0.03	0.01	-2.51^*^			
Ostracoda	Intercept	127.03	41.38	3.07^*^^*^	13.27	326.51	^*^
	Season spring	-0.97	0.42	-2.30^*^			
	Season winter	-0.79	0.38	-2.09^*^			
	Salinity	-3.19	1.06	-3.02^*^^*^			
Chaetognatha	Intercept	1.19	0.20	5.99^*^^*^^*^	19.62	298.05	^*^^*^
	Season spring	0.86	0.28	3.06^*^^*^			

^*^: *P* < 0.05; ^*^^*^: *P* < 0.01; ^*^^*^^*^: *P* < 0.001. For ANOVA, null vs final model *P* value < 0.05 indicates that the final model is better than the null model.

## DISCUSSION

The study of the spatial and seasonal variability of coastal zooplankton communities, and their relation with environmental conditions, improves our understanding on the function of a distinct coastal ecosystem in an ultra-oligotrophic environment. This is the first attempt to characterize the zooplankton dynamics in the coastal waters of Cyprus, over a 12-month period time series constituting a comprehensive study, in terms of the sampling effort, as 66 MZ samples were collected in a complete year cycle, the taxonomic analysis, which revealed a high number of MZ taxa, the majority of which are new references to Cyprus coastal waters, and the phenology of MZ.

The previous attempts investigating MZ in the coastal waters of Cyprus (Hannides et al., 2015b; Vasilopoulou et al., 2022), were carried out mainly with seasonal sampling, but with gaps among seasons and stations. The sampling locations, of the current study, which were selected based on the differences in temperature (Akrotiri and Kato Pyrgos) and anthropogenic pressures gradient (Vasiliko) are geographically in different locations from the sampling stations of the previous studies. Moreover, the monthly sampling of MZ in our work was carried out using a WP2 closing net, by conducting two vertical tows, 0–50 m and 50–100 m, in AKR and PYR, investigating the vertical distribution of MZ, while Hannides et al. (2015b) carried out vertical tows with an average tow depth ~ 100 m and Vasilopoulou et al. (2022) carried out only one vertical tow (0–50 m), overall. These studies collectively provide valuable information and enhance our knowledge on MZ dynamics in the coastal waters of Cyprus.

The recommended use of a 200-μm net (UNESCO, 1968) for zooplankton sampling, that was used the current study leads to a biased view of the taxonomy and abundance of the copepod community (Gallienne and Robins, 2001). Sampling with finer mesh nets than the standard 200 μm, has revealed that biomass and abundance can increase by 2- to 20-fold when the smaller metazooplankters (∼50–200 μm) are considered (Zervoudaki et al., 2006; Zervoudaki et al., 2020). Nevertheless, the use of 200—μm mesh nets, which is commonly used in the majority of zooplankton studies in the Mediterranean allows the comparison among other studies conducted in the area (Hannides et al., 2015b; Terbiyik-Kurt and Polat, 2015; Vasilopoulou et al., 2022;) as well as with other geographical regions, e.g. Aegean (Isari et al., 2006) and Western Mediterranean (Mazzocchi et al., 2011).

The difference in temperature and salinity that was observed in the surface layer of Kato Pyrgos in comparison with Akrotiri and Vasiliko (Fig. 2) during the summer period might be due to coastal upwelling in the south of Cyprus during summer, caused by persistent westerly winds that affect the near-surface layers (Mauri et al., 2019; Zodiatis et al., 2008), which caused the decreased temperature at the southern stations. The concentration of Chl-a exhibited a clear increasing gradient proportional to increasing depth overall, as the maximum values of Chl-a were recorded at 75 and 100 m depth (Figs 2 and 6). This is consistent with the deep chlorophyll maximum (DCM) recorded in the Levantine, where the vertical distribution of Chl-a ranges between 80 and 120 m depth, being shallower in the nearshore areas (Berman et al., 1986; Herut et al., 2000). The above two parameters (temperature and Chl-a) also identified as key factors for the distribution of MZ in the coastal waters of Cyprus and the separation of winter stations from the other with three seasons (winter community and spring–summer-autumn community) (Fig. 8) This is also supported by the “indispecies” analysis, as a higher number of taxa act as indicators for winter in comparison with the other three seasons, which have common indicator taxa. These results could be attributed mostly to the mixing period (December—April) and the stratification period (May–December) and, respectively (Figs 2 and 3).

The nutrient regime did not show a clear pattern and the concentrations of nutrients in the study area were not typical of the open waters of the Levantine basin (Figs 2 and 3). N:P ratio was lower than the Redfield ratio of 16, and the PO_4_^−3^ concentrations were particularly high in winter, spring and autumn (Figs 2 and 3) as it has been also reported by the ongoing monitoring programs in Cyprus waters (DFMR, internal reports). Similar values of PO_4_^−3^ were also found in Vasiliko Bay in July 2008 (Tsagaraki et al., 2013) and in other coastal areas of the broader area of Levantine basin, such as in Israel (Kress et al., 2019), as well as low N:P values similarly to present study. In addition, low values of N:P were found in the Mersin Bay (Yücel, 2018). According to Moksness et al. (2013) the area of Vasiliko is one of the most impacted coastal areas in Cyprus. Among the industries located there, are a cement production factory, and an abandoned chemical fertilizers industry, a power station, the largest desalination plant of the island and the majority of the aquacultures in Cyprus. Similarly, the area of Kato Pyrgos, is not serviced by a central sewerage system. The aforementioned factors are highly likely to increase the local nutrient concentration of Vasiliko and Kato Pyrgos. In oligotrophic environments, where small-sized cells are dominant, nutrient addition is not followed by a relative response of phytoplankton abundance, as was observed in fish farms in the eastern Mediterranean (Pitta et al., 2009). This is mainly attributed to the “empowerment” of the microbial loop, which engages grazing by microzooplankton, and specifically to heterotrophic flagellates and ciliates, before it becomes available to consumers (zooplankton) (Suthers and Rissik, 2009). This is probably the case for VAS1 in spring where a bloom event of *Isias clavipes* was observed and increase abnormally the total abundance and biomass of the station (Fig. 5). This species demonstrated also high abundance in VAS2 the same period. This species is well adapted in fluctuating environments (Miloslavić and Lučić, 2015) being rare in the Levantine Sea and should be an indicator of the Atlantic current in the Mediterranean Sea (Lakkis, 1990). Moreover, the fact that VAS1 and VAS2 are shallower stations in comparison to AKR and PYR, possibly contributed to the increase of population of *I. clavipes*, as this species seems to prefer swallower waters. The bloom event of *Isias clavipes* it seems to be the reason for the higher value of abundance that was ever reported in the coastal of Cyprus, compared to previous studies (Hannides et al., 2015b; Vasilopoulou et al., 2022). In addition to that event, VAS1 and VAS2 generally presented higher MZ abundance in the layer 0–50 m, compared to AKR and PYR (Fig. 4). PYR located in the north of the island showed a different seasonal pattern of total abundance from the southern stations; lowest values in PYR occurred in summer, whereas the lowest mean total abundance for AKR, VAS1 and VAS2 occurred in winter. This could also be triggered by the coastal upwelling present during the summer, southern of Cyprus, and/or the proportion of nanophytoplankton and picophytoplankton, among them. Demetriou et al. (2022), based on phytoplankton sample analysis from the same stations, reported that during the stratified period (May–December) in southern stations AKR and VAS, the percentage of nanophytoplankton was higher, thus nanophytoplankton dominated over picophytoplankton. In general, it has been found that picoplankton dominates the eastern Mediterranean surface layers during most of the year (Zohary et al.,1998; Tanaka et al., 2007) with the exception of the dynamic mesoscale structures, where nanophytoplankton seems to be the dominant size class (Psarra et al., 2005; Tanaka et al., 2007).

In general, the bulk of epipelagic MZ in the open MS is concentrated in the upper 100 m layer where they play a major role in biological processes (Longhurst and Harrison, 1989; Siokou-Frangou et al., 2010) and sharply decreases below this depth (Mazzocchi et al., 1997; Scotto di Carlo et al., 1984).

The Goodness of fit in combination with the Permanova test (SVII) showed that there is a weak, but statistically significant correlation between MZ communities and sampling season. This is supported also by the “indispecies” analysis that revealed different indicator taxa for each season, but not for summer (Table II). Although, a spatial pattern regarding the total MZ abundance was observed, in summer, between southern and north stations, due to annual observed coastal upwelling, “indispecies” analysis didn’t identify a specific spatial pattern regarding MZ taxa, in general. Similar results were reported by Vasilopoulou et al., (2022), regarding the spatial distribution of MZ taxa. This is probably a result of the prevailing circulation in the waters of Cyprus and the open sea influence, the Mid-Mediterranean Jet, flowing north along the western coasts and the Asia Minor Current, flowing along the southern coasts (Zodiatis et al., 2008), that seems to prevail the influence from inland inputs, which are limited. The number of taxa found here, showed an increasing trend with depth in both stations of AKR and PYR, all over the year. Other studies in the Mediterranean have also detected such a vertical differentiation in species composition (Isari et al., 2006; Scotto di Carlo et al., 1984; Siokou-Frangou et al., 1997). The difference of the 0–50 m community from that of 50–100 m can be possibly attributed to the fact that the DCM occurred in the depth of 75–100 m and the long stratification period that occurred from May to December. This difference was more evident in spring, in PYR, where the water column stratification appeared, and in autumn in AKR. The oscillation of the number of copepod taxa in this study followed the seasonal and bathymetric trend of the total MZ taxa overall.

MZ biomass in the present study was among the lowest reported in the Mediterranean Sea (Siokou-Frangou et al., 2010) and presented similar range with the one reported by Hannides et al. (2015b). Significant fluctuations of biomass between layers in winter (e.g. in PYR—Fig. 4) were probably due to the higher abundance in the layer of 0–50 m of large sized copepods *Clausocalanus lividus, C. mastigophorus* and medium sized *C. jobei* as well as the higher abundance of *Clausocalanus* spp. juveniles and *C. paululus.* On the other hand, the lowest biomass in both VAS1 and VAS2 observed in August indicate that the structure of MZ community is formed by smaller copepods, as the mean abundance in the summer months at these stations is higher in comparison to winter (Fig. 4).

Copepoda were by far the most important taxon of zooplankton in the area (Fig. 4). Their dominance in the coastal waters of Cyprus is in agreement to the trend observed in all the Mediterranean Sea, as they represent the major group both in terms of abundance and biomass (Siokou-Frankou et al., 2010), and the open waters of the Levantine basin (Mazzocchi et al., 1997). The dominance of copepods in the total MZ abundance agrees also with the previous studies conducted on the coasts of Cyprus by Hannides et al. (2015b) and Vasilopoulou et al. (2022). The relative abundance of the other groups of MZ in this study followed similar trend observed in other areas of the Mediterranean, e.g. ostracods increase gradually with depth and vary from ∼2% in the upper 50 m to ∼11% in the 200–300 m layer (Siokou-Frankou et al. 2010). Similar relative abundance for ostracods was observed in the present study (2%), as well as the proportional increase of their abundance with depth. A similar increasing trend of ostracods with depth was found also by Isari et al. (2006) in north–eastern Aegean Sea. Brautovic et al. (2006) found that the highest abundance of ostracods occurred during the cold period something that might be related to the minimum abundance of potential predators (gelatinous and semi-gelatinous zooplankton). In the present study, ostracods exhibited higher abundance in AKR compared to PYR during summer when temperature was lower. Moreover, the higher occurrence of this group in the deeper layer might be related to the lower abundance of predators in this layer, such as Cnidaria that were more abundant in the upper layer. Appendicularia, which present high population growth rate under favorable conditions (Gorsky and Palazzoli, 1989), were found in higher abundance in winter and summer in AKR (attributed to the summer upwelling in the southern area of Cyprus). In addition, Appendicularia presented the highest contribution to the total abundance in winter in Naples, when the annual minimum in MZ standing stocks is always recorded (Ribera d’Alcala et al., 2004; Mazzocchi et al., 2011). Appendicularia seem to correlate with winter phytoplankton bloom better than copepods (Ribera d’Alcala et al., 2004; Mazzocchi et al., 2011). Similarly, Chl-a was positively correlated with Appedicularia in the present study (Table I). The increase of appendicularian abundance with depth for most of the year in the present study, along with the increasing trend of oncaeid abundance with depth, agrees with the correlation between these groups found by Mazzocchi et al. (2011). It seems that in oligotrophic waters, oncaeids may benefit from food items (sinking aggregates) originating from discharged appendicularian houses (Ohtsuka et al., 1996), and are not accessible to calanoids (Mazzocchi et al., 2011). The seasonal distribution of Cladocera in this study agrees to Ribera d’Alcala et al. (2004), who reported similar seasonal variation for Cladocera. According to Siokou-Frangou (1996) and Mazzocchi et al. (2011), Cladocera in the summer and Appendicularia mainly in winter and spring, exhibit strong population outbursts for short time periods when conditions are favorable. Moreover, Isari et al. (2006) and Ramfos et al. (2006) reported high cladocerans abundance in the layer of 0–50 m. These groups seem to play an important role in marine ecosystems as they are able to feed directly on components of the microbial food web (Sommer et al., 2002), thus contributing to a more efficient transfer of energy towards higher trophic levels (Siokou-Frangou et al., 2002).

Calanoida and Cyclopoida were by far the most dominant groups of copepods in the present study, constituting significant proportion (2/3 and 1/3, respectively) of the total copepod abundance. Similar results were found in other studies in the Levantine basin (Mazzocchi et al., 1997; Pasternak et al., 2005). Calanoida, exhibited a decreasing pattern with depth. This is in agreement with the general vertical pattern of zooplankton found in Levantine basin (Mazzocchi et al., 1997). Clausocalanidae was the dominant Family of copepods in this study, constituting more than one third of their total abundance. *C. paululus,* the smallest in size representative of Clausocalanidae has important contribution to this Family and to the total abundance in general (Table SIII). It was found that is the characteristic species of the subsurface assemblage in the Ionian Sea, the Cretan Passage and the central Levantine Sea (Mazzocchi et al., 1997). *C. paululus*, demonstrated a decreasing seasonal pattern from winter to early autumn, similar to previous studies (Peralba and Mazzocchi, 2004) and it is referred as a winter species (Mazzocchi et al., 2011). Similar observations were made also in previous studies for *C. furcatus* showing high abundance in spring (Mazzocchi et al., 2011) and autumn (Siokou-Frangou et al., 1997). In addition, *C. furcatus, T. stylifera* and the cladoceran *Evadne spinifera* presented lower abundances in the layer of 50–100 m, as it has been also observed in other studies (Isari et al., 2006; Ramfos et al., 2006). On the other hand, *Haloptilus longicornis* and *Mesocalanus tenuicornis* exhibited an increasing trend in the deeper layer, in agreement with observations found by Mazzocchi et al. (2011) and Ramfos et al. (2006), respectively.

The Eastern Levantine Basin is heavily affected by species introductions, through the Suez Canal. Despite that, the analysis of the temporal trend of alien species belonging to small crustaceans (e.g. copepods, mysids, cumaceans and others) is for the moment unreliable, because the information is scarce, and sometimes too recent (Zenetos et al., 2012). In our case, the high sampling effort revealed five alien species (*Calanopia elliptica, Centropages furcatus, Euchaeta concinna, Triconia rufa* and *Triconia hawii*), out of the 33 introduced copepoda taxa reported in the Eastern Mediterranean (Zenetos et al., 2010). As far as we know, these alien species are first reports in Cyprus and Levantine basin (Zenetos et al., 2010; Zenetos and Galanidi, 2020). It is believed that the relatively few planktonic migrants in the area will increase over time, due to the decreasing of the Nile fresh water inflow into the Mediterranean and lower salinity in the Bitter lakes (Halim, 1990; Zenetos et al., 2006).

The contribution of cyclopoids in the Aegean Sea, as well as in the neighboring Ionian and Levantine Seas, has also been reported in previous studies (Siokou-Frangou et al., 1997; Zervoudaki et al., 2006). In addition, the increasing trend of Cyclopoida with depth was also found in South Aegean Sea (Siokou et al., 2013). The high abundance of egg-carrying genera indicates a successful life strategy in this oligotrophic environment. Their lower egg mortality and metabolic demands, behavioral adaptations to reduce predation, and the development of various feeding strategies undoubtedly contribute to maintain their prominent appearance to the oligotrophic environment of the Aegean Sea (Zervoudaki et al., 2006) and the ultra-oligotrophic Levantine basin. *O. plumifera,* the most abundant species of *Oithona* species in this study presented similar seasonal and vertical distribution to previous studies (Siokou-Frangou et al., 1997; Ramfos et al., 2006; Siokou-Frankou et al., 2010; Vasilopoulou et al., 2022). The increasing pattern of Oncaeids with depth was also in agreement with previous studies (Ramfos et al., 2006; Siokou et al., 2013).

## CONCLUSIONS

To the best of our knowledge, the present study is the first attempt to provide information on the zooplankton monthly and seasonal succession in Cyprus, over a 12-month period time series. The difference of temperature during summer, between the south (AKR, VAS1 and VAS2) and north (PYR) sampling stations, due to the combination of upwelling and advection from the Rhodes Gyre, seems to control the food supply, hence offers favorable feeding conditions to zooplankton organisms, enhancing their numbers. Anthropogenic pressures also seem to affect MZ dynamics, as the proximity of the VAS1 station to a fish-farm had a positive effect on the abundance and biomass of MZ. The importance of smaller species (e.g. *C. paululus*) and stages (e.g. *Clausocalanus*, *Oithona* and *Corycaeus* spp. juveniles) in the composition and structure of Cyprus coastal waters’ copepod community is also revealed. These species have been reported to be important in low Chl-a environments, where the relative size of primary consumers is expected to be smaller and the microbial components dominant (Calbet et al., 2000; Zervoudaki et al., 2006). This baseline study paves the way for further investigation of the elements of marine food webs through a future long-term monitoring. Such future work will be paramount for developing a better understanding of zooplankton dynamics and phenology in the coastal waters of Cyprus, with implications for the evaluation of climate change effects (rising temperatures, ocean acidification) and other human-driven environmental perturbations, the implementation of the Marine Strategy Framework Directive (MSFD) and the marine environment in general.

## Supplementary Material

G_Fyttis_et_al_Supplementary_material_31_10_2022_clean_fbac075

## Data Availability

The data underlying this article are available in the article and in its online supplementary material.
