# Supplementary material for: Annual cycle of mesozooplankton at the coastal waters of Cyprus (Eastern Levantine basin)
Source: J Plankton Res. 2023 Jan 20;45(2):291–311. doi: 10.1093/plankt/fbac075 (PMC10066811; doi:10.1093/plankt/fbac075)
Supplement: G_Fyttis_et_al_Supplementary_material_31_10_2022_clean_fbac075 [file g_fyttis_et_al_supplementary_material_31_10_2022_clean_fbac075.docx]

| Table SI. Summary statistics (min, max, average ± SDEV) of MZ abundance (ind m^-3^) overall. | | | | | | | | | |
| --- | --- | --- | --- | --- | --- | --- | --- | --- | --- |
|  | **Total MZ** | **AKR** | | | **PYR** | | | **VAS1** | **VAS2** |
|  |  | **0-50m** | **50-100m** | **Total** | **0-50m** | **50-100m** | **Total** | **50m** | **50m** |
| **Min** | 106 | 231 | 106 | 106 | 164 | 122 | 122 | 234 | 362 |
| **Max** | 1,257 | 704 | 509 | 704 | 504 | 758 | 763 | 1,257 | 685 |
| **Average** | 382 ± 171.68 | 380 ± 131.30 | 321 ± 120.41 | 351 ± 129.28 | 330 ± 96.54 | 347 ± 165.06 | 339 ± 135.47 | 470 ± 287.47 | 466 ± 97.83 |

| Table SII. Summary statistics (min, max, average ± SDEV) of MZ biomass overall. | | | | | | | | | |
| --- | --- | --- | --- | --- | --- | --- | --- | --- | --- |
|  | **Total MZ** | **AKR** | | | **PYR** | | | **VAS1** | **VAS2** |
|  |  | **0-50m** | **50-100m** | **Total** | **0-50m** | **50-100m** | **Total** | **50m** | **50m** |
| **Min (mg mˉ³)** | 0.39 | 0.86 | 0.94 | 0.86 | 0.39 | 0.71 | 0.71 | 1.57 | 1.25 |
| **Max (mg mˉ³)** | 4.55 | 3.29 | 2.20 | 3.29 | 4.07 | 2.67 | 4.07 | 4.55 | 2.82 |
| **Average (mg mˉ³)** | 1.81 ± 0.80 | 1.74 ± 0.56 | 1.66 ± 0.43 | 1.66± 0.56 | 1.49 ± 0.93 | 1.44 ± 0.55 | 1.47 ± 0.75 | 2.67 ± 0.99 | 2.11 ± 0.44 |

| Table SIII. Summary statistics (min, max and average) for the abundance (ind. m^-3^) of all recorded MZ taxa overall. | | |  |  |  |  |  |
| --- | --- | --- | --- | --- | --- | --- | --- |
| **Mesozooplankton taxa** | Mean abundance (ind. m^-3^) | Min abundance (ind. m^-3^) | Max abundance (ind. m^-3^) |  |  |  |  |
| *Evadne spinifera* P.E. Müller, 1867 | 2.62 | 0.00 | 40.00 |  |  |  |  |
| *Pseudevadne tergestina* Claus, 1877 | 1.72 | 0.00 | 23.53 |  |  |  |  |
| *Podon* sp. Lilljeborg, 1853 | 0.04 | 0.00 | 2.35 |  |  |  |  |
| *Acartia adriatica* Steuer, 1910 | 1.05 | 0.00 | 40.78 |  |  |  |  |
| *Acartia clausi* Giesbrecht, 1889 | 0.06 | 0.00 | 0.78 |  |  |  |  |
| *Acartia negligens* Dana, 1849 | 4.60 | 0.00 | 26.67 |  |  |  |  |
| *Acartia* spp. juveniles | 3.69 | 0.00 | 31.37 |  |  |  |  |
| *Aetideus acutus* Farran, 1929 | 0.04 | 0.00 | 1.57 |  |  |  |  |
| *Aetideus* spp. juveniles | 0.01 | 0.00 | 0.78 |  |  |  |  |
| *Haloptilus longicornis* Claus, 1863 | 5.77 | 0.00 | 36.08 |  |  |  |  |
| *Haloptilus* spp. juveniles | 0.14 | 0.00 | 3.14 |  |  |  |  |
| Calanidae Dana, 1849 | 0.97 | 0.00 | 7.06 |  |  |  |  |
| *Mesocalanus tenuicornis* Dana, 1849 | 2.96 | 0.00 | 18.82 |  |  |  |  |
| *Nannocalanus minor* Claus, 1863 | 2.94 | 0.00 | 17.25 |  |  |  |  |
| *Neocalanus gracilis* Dana, 1852 | 0.32 | 0.00 | 14.12 |  |  |  |  |
| *Candacia bispinosa* Claus, 1863 | 0.21 | 0.00 | 3.92 |  |  |  |  |
| *Candacia curta* Dana, 1849 | 0.04 | 0.00 | 0.78 |  |  |  |  |
| *Candacia simplex* Giesbrecht, 1889 | 0.17 | 0.00 | 4.71 |  |  |  |  |
| *Candacia varicans* Giesbrecht, 1893 | 0.02 | 0.00 | 0.78 |  |  |  |  |
| *Candacia* spp. | 0.01 | 0.00 | 0.78 |  |  |  |  |
| *Candacia* spp. juveniles | 0.62 | 0.00 | 3.92 |  |  |  |  |
| *Centropages bradyi* Wheeler, 1900 | 0.05 | 0.00 | 2.35 |  |  |  |  |
| *Centropages furcatus* Dana, 1849 | 0.01 | 0.00 | 0.78 |  |  |  |  |
| *Centropages kroyeri* Giesbrecht, 1893 | 0.58 | 0.00 | 22.75 |  |  |  |  |
| *Centropages violaceus* Claus, 1863 | 0.59 | 0.00 | 5.49 |  |  |  |  |
| *Isias clavipes* Boeck, 1865 | 9.28 | 0.00 | 520.78 |  |  |  |  |
| *Clausocalanus arcuicornis* Dana, 1849 | 0.81 | 0.00 | 7.84 |  |  |  |  |
| *Clausocalanus furcatus* Brady, 1883 | 16.01 | 0.00 | 116.86 |  |  |  |  |
| *Clausocalanus jobei* Frost & Fleminger, 1968 | 1.73 | 0.00 | 13.33 |  |  |  |  |
| *Clausocalanus lividus* Frost & Fleminger, 1968 | 2.59 | 0.00 | 47.84 |  |  |  |  |
| *Clausocalanus mastigophorus* Claus, 1863 | 1.78 | 0.00 | 19.61 |  |  |  |  |
| *Clausocalanus parapergens* Frost & Fleminger, 1968 | 2.47 | 0.00 | 24.31 |  |  |  |  |
| *Clausocalanus paululus* Farran, 1926 | 20.46 | 0.00 | 97.65 |  |  |  |  |
| *Clausocalanus pergens* Farran, 1926 | 0.92 | 0.00 | 10.20 |  |  |  |  |
| *Clausocalanus* spp. males | 0.27 | 0.00 | 7.84 |  |  |  |  |
| *Clausocalanus* spp. juveniles | 62.38 | 4.71 | 292.55 |  |  |  |  |
| *Ctenocalanus vanus* Giesbrecht, 1888 | 0.05 | 0.00 | 0.78 |  |  |  |  |
| *Ctenocalanus* spp. juveniles | 0.11 | 0.00 | 5.49 |  |  |  |  |
| *Diaixis pygmaea* Scott T., 1894 | 0.80 | 0.00 | 20.39 |  |  |  |  |
| *Pareucalanus attenuatus* Dana, 1849 | 0.10 | 0.00 | 1.57 |  |  |  |  |
| *Euchaeta concinna* Dana, 1849 | 0.02 | 0.00 | 0.78 |  |  |  |  |
| *Euchaeta marina* Prestandrea, 1833 | 0.14 | 0.00 | 3.14 |  |  |  |  |
| *Euchaeta* spp. juveniles | 0.40 | 0.00 | 3.14 |  |  |  |  |
| *Heterorhabdus papilliger* Claus, 1863 | 0.59 | 0.00 | 4.71 |  |  |  |  |
| *Lucicutia clausi* Giesbrecht, 1889 | 0.01 | 0.00 | 0.78 |  |  |  |  |
| *Lucicutia flavicornis* Claus, 1863 | 2.48 | 0.00 | 14.90 |  |  |  |  |
| *Lucicutia gaussae* Grice, 1963 | 0.65 | 0.00 | 6.27 |  |  |  |  |
| *Lucicutia gemina* Farran, 1926 | 0.47 | 0.00 | 4.71 |  |  |  |  |
| *Lucicutia longicornis* Giesbrecht, 1889 | 0.01 | 0.00 | 0.78 |  |  |  |  |
| *Lucicutia* spp. | 0.04 | 0.00 | 1.57 |  |  |  |  |
| *Lucicutia* spp. males | 0.02 | 0.00 | 0.78 |  |  |  |  |
| Lucicutia spp. juveniles | 2.93 | 0.00 | 13.33 |  |  |  |  |
| *Pleuromamma abdominalis* Lubbock, 1856 | 0.07 | 0.00 | 1.57 |  |  |  |  |
| *Pleuromamma gracilis* Claus, 1863 | 0.64 | 0.00 | 4.71 |  |  |  |  |
| *Pleuromamma xiphias* Giesbrecht, 1889 | 0.01 | 0.00 | 0.78 |  |  |  |  |
| *Pleuromamma* spp. juveniles | 1.11 | 0.00 | 14.12 |  |  |  |  |
| *Calocalanus contractus* Farran, 1926 | 0.40 | 0.00 | 4.71 |  |  |  |  |
| *Calocalanus latus* Shmeleva, 1968 | 0.02 | 0.00 | 1.57 |  |  |  |  |
| *Calocalanus neptunus* Shmeleva, 1965 | 0.20 | 0.00 | 2.35 |  |  |  |  |
| *Calocalanus ovalis* Shmeleva, 1965 | 0.01 | 0.00 | 0.78 |  |  |  |  |
| *Calocalanus pavo* Dana, 1852 | 2.29 | 0.00 | 18.04 |  |  |  |  |
| *Calocalanus pavonicus* Farran, 1936 | 10.66 | 0.00 | 66.67 |  |  |  |  |
| *Calocalanus plumatus* Shmeleva, 1965 | 2.59 | 0.00 | 15.69 |  |  |  |  |
| *Calocalanus plumulosus* Claus, 1863 | 1.33 | 0.00 | 6.27 |  |  |  |  |
| *Calocalanus styliremis* Giesbrecht, 1888 | 5.40 | 0.00 | 32.16 |  |  |  |  |
| *Calocalanus* spp. juveniles | 0.45 | 0.00 | 8.63 |  |  |  |  |
| *Mecynocera clausi* Thompson I.C., 1888 | 9.27 | 0.00 | 31.37 |  |  |  |  |
| *Mecynocera gracilis* Tanaka, 1956 | 0.07 | 0.00 | 2.35 |  |  |  |  |
| *Paracalanus denudatus* Sewell, 1929 | 9.49 | 0.00 | 43.92 |  |  |  |  |
| *Paracalanus nanus* Sars G.O., 1925 | 0.65 | 0.00 | 9.94 |  |  |  |  |
| *Paracalanus parvus* Claus, 1863 | 0.46 | 0.00 | 5.49 |  |  |  |  |
| *Paracalanus* spp. | 0.01 | 0.00 | 0.78 |  |  |  |  |
| *Paracalanus* spp. juveniles | 0.54 | 0.00 | 3.92 |  |  |  |  |
| *Phaenna spinifera* Claus, 1863 | 0.01 | 0.00 | 0.78 |  |  |  |  |
| *Calanopia elliptica* Dana, 1849 | 0.03 | 0.00 | 1.57 |  |  |  |  |
| *Calanopia metu* Uysal & Shmeleva, 2004 | 0.04 | 0.00 | 2.35 |  |  |  |  |
| *Pontellina plumata* Dana, 1849 | 0.01 | 0.00 | 0.78 |  |  |  |  |
| *Amallothrix tenuiserrata* Giesbrecht, 1893 | 0.39 | 0.00 | 4.71 |  |  |  |  |
| *Scaphocalanus curtus* Farran, 1926 | 0.15 | 0.00 | 3.14 |  |  |  |  |
| *Scaphocalanus* spp. | 0.01 | 0.00 | 0.78 |  |  |  |  |
| *Scaphocalanus* spp. juveniles | 0.01 | 0.00 | 0.78 |  |  |  |  |
| *Scolecithricella dentata* Giesbrecht, 1893 | 0.17 | 0.00 | 1.57 |  |  |  |  |
| *Scolecithricella/Amallothrix* spp. juveniles | 0.97 | 0.00 | 4.71 |  |  |  |  |
| *Scolecithrix bradyi* Giesbrecht, 1888 | 0.55 | 0.00 | 11.76 |  |  |  |  |
| *Scolecithrix* spp. juveniles | 0.28 | 0.00 | 5.49 |  |  |  |  |
| *Scolecithrix* spp. males | 0.01 | 0.00 | 0.78 |  |  |  |  |
| *Temora stylifera* Dana, 1849 | 11.75 | 0.00 | 65.10 |  |  |  |  |
| *Agetus flaccus* Giesbrecht, 1891 | 1.50 | 0.00 | 8.63 |  |  |  |  |
| *Agetus limbatus* Brady, 1883 | 4.30 | 0.00 | 26.67 |  |  |  |  |
| *Agetus typicus* Krøyer, 1849 | 4.41 | 0.00 | 21.96 |  |  |  |  |
| *Corycaeus clausi* Dahl F., 1894 | 1.56 | 0.00 | 9.41 |  |  |  |  |
| *Corycaeus speciosus* Dana, 1849 | 1.51 | 0.00 | 16.47 |  |  |  |  |
| *Ditrichocorycaeus anglicus* Lubbock, 1857 | 0.01 | 0.00 | 0.78 |  |  |  |  |
| *Ditrichocorycaeus brehmi* Steuer, 1910 | 0.39 | 0.00 | 3.92 |  |  |  |  |
| *Ditrichocorycaeus mininus indicus* Dahl, 1912 | 0.90 | 0.00 | 5.49 |  |  |  |  |
| *Onychocorycaeus giesbrechti* Dahl F., 1894 | 0.70 | 0.00 | 7.84 |  |  |  |  |
| *Onychocorycaeus latus* Dana, 1849 | 0.22 | 0.00 | 8.63 |  |  |  |  |
| *Onychocorycaeus ovalis* Claus, 1863 | 0.61 | 0.00 | 4.71 |  |  |  |  |
| *Urocorycaeus furcifer* Claus, 1863 | 1.41 | 0.00 | 11.76 |  |  |  |  |
| *Corycaeus* spp. males | 0.05 | 0.00 | 3.14 |  |  |  |  |
| *Corycaeus* spp. juveniles | 9.07 | 0.78 | 28.24 |  |  |  |  |
| *Farranula rostrata* Claus, 1863 | 17.93 | 1.57 | 44.71 |  |  |  |  |
| *Lubbockia squillimana* Claus, 1863 | 0.23 | 0.00 | 2.35 |  |  |  |  |
| *Oithona atlantica* Farran, 1908 | 1.88 | 0.00 | 19.61 |  |  |  |  |
| *Oithona decipiens* Farran, 1913 | 0.02 | 0.00 | 0.78 |  |  |  |  |
| *Oithona longispina* Nishida, Tanaka & Omori, 1977 | 2.75 | 0.00 | 10.98 |  |  |  |  |
| *Oithona nana* Giesbrecht, 1893 | 0.05 | 0.00 | 1.57 |  |  |  |  |
| *Oithona plumifera* Baird, 1843 | 5.49 | 0.00 | 52.55 |  |  |  |  |
| *Oithona setigera* Dana, 1849 | 1.31 | 0.00 | 12.55 |  |  |  |  |
| *Oithona similis* Claus, 1866 | 0.07 | 0.00 | 0.78 |  |  |  |  |
| *Oithona tenuis* Rosendorn, 1917 | 2.05 | 0.00 | 10.98 |  |  |  |  |
| *Oithona vivida* Farran, 1913 | 0.59 | 0.00 | 3.92 |  |  |  |  |
| *Oithona* spp. | 0.04 | 0.00 | 1.57 |  |  |  |  |
| *Oithona* spp. males | 0.38 | 0.00 | 3.53 |  |  |  |  |
| *Oithona* spp. juveniles | 20.29 | 0.00 | 57.25 |  |  |  |  |
| Unknown cyclopodia | 0.01 | 0.00 | 0.39 |  |  |  |  |
| *Oncaea curta* Sars G.O., 1916 | 0.02 | 0.00 | 0.78 |  |  |  |  |
| *Oncaea media* Giesbrecht, 1891 | 1.61 | 0.00 | 12.55 |  |  |  |  |
| *Oncaea mediterranea* Claus, 1863 | 2.42 | 0.00 | 10.20 |  |  |  |  |
| *Oncaea scottodicarloi* Heron & Bradford-Grieve, 1995 | 5.27 | 0.00 | 29.02 |  |  |  |  |
| *Oncaea venusta* Philippi, 1843 | 0.31 | 0.00 | 3.92 |  |  |  |  |
| *Triconia conifera* Giesbrecht, 1891 | 0.39 | 0.00 | 3.92 |  |  |  |  |
| *Triconia dentipes* Giesbrecht, 1891 | 0.01 | 0.00 | 0.78 |  |  |  |  |
| *Triconia furcula* Farran, 1936 | 0.01 | 0.00 | 0.78 |  |  |  |  |
| *Triconia hawii* Böttger-Schnack & Boxshall, 1990 | 0.62 | 0.00 | 9.41 |  |  |  |  |
| *Triconia rufa* Boxshall & Böttger, 1987 | 0.01 | 0.00 | 0.39 |  |  |  |  |
| *Oncaea* spp. males | 3.91 | 0.00 | 23.53 |  |  |  |  |
| *Oncaea* spp. juveniles | 1.77 | 0.00 | 15.69 |  |  |  |  |
| *Copilia quadrata* Dana, 1849 | 0.33 | 0.00 | 2.35 |  |  |  |  |
| *Copilia* spp. | 0.01 | 0.00 | 0.78 |  |  |  |  |
| *Sapphirina angusta* Dana, 1849 | 0.02 | 0.00 | 1.57 |  |  |  |  |
| *Sapphirina auronitens* Claus, 1863 | 0.01 | 0.00 | 0.78 |  |  |  |  |
| *Sapphirina lactens* Giesbrecht, 1893 | 0.01 | 0.00 | 0.78 |  |  |  |  |
| *Sapphirina metallina* Dana, 1849 | 0.70 | 0.00 | 7.06 |  |  |  |  |
| *Sapphirina nigromaculata* Claus, 1863 | 0.04 | 0.00 | 0.78 |  |  |  |  |
| *Sapphirina opalina* Dana, 1849 | 0.02 | 0.00 | 0.78 |  |  |  |  |
| *Sapphirina* spp. | 0.13 | 0.00 | 1.57 |  |  |  |  |
| *Vettoria granulosa* Giesbrecht, 1891 | 0.02 | 0.00 | 1.57 |  |  |  |  |
| *Vettoria parva* Farran, 1936 | 0.25 | 0.00 | 1.57 |  |  |  |  |
| *Microsetella norvegica* Boeck, 1865 | 0.02 | 0.00 | 0.78 |  |  |  |  |
| *Macrosetella gracilis* Dana, 1846 | 0.07 | 0.00 | 1.57 |  |  |  |  |
| *Clytemnestra* spp. *juveniles* | 0.01 | 0.00 | 0.78 |  |  |  |  |
| *Clytemnestra scutellata* Dana, 1847 | 0.04 | 0.00 | 1.57 |  |  |  |  |
| *Clytemnestra* spp. | 0.04 | 0.00 | 1.57 |  |  |  |  |
| *Clytemnestra/Goniopsyllus* spp. juveniles | 0.01 | 0.00 | 0.78 |  |  |  |  |
| *Euterpina acutifrons* Dana, 1847 | 0.02 | 0.00 | 0.78 |  |  |  |  |
| *Monstrilla* spp. | 0.01 | 0.00 | 0.39 |  |  |  |  |
| Unknown Siphonostomatoida | 0.01 | 0.00 | 0.78 |  |  |  |  |
| Amphipoda | 0.20 | 0.00 | 2.35 |  |  |  |  |
| *Rhabdosoma minor* Fage, 1954 | 0.01 | 0.00 | 0.78 |  |  |  |  |
| Cirripedia | 0.14 | 0.00 | 1.57 |  |  |  |  |
| Uknown Crustacea | 0.07 | 0.00 | 1.57 |  |  |  |  |
| Decapoda | 0.57 | 0.00 | 25.88 |  |  |  |  |
| Decapoda larvae | 6.73 | 0.00 | 43.92 |  |  |  |  |
| *Lucifer typus* H. Milne Edwards, 1837 | 0.02 | 0.00 | 0.78 |  |  |  |  |
| *Scyllarus arctus* larvae Linnaeus, 1758 | 0.02 | 0.00 | 0.78 |  |  |  |  |
| *Sergestes* spp. | 0.01 | 0.00 | 0.78 |  |  |  |  |
| Euphausiacea | 0.04 | 0.00 | 1.57 |  |  |  |  |
| Euphausiacea larvae | 0.06 | 0.00 | 1.57 |  |  |  |  |
| Mysida | 0.52 | 0.00 | 18.04 |  |  |  |  |
| Ostracoda | 7.23 | 0.00 | 33.73 |  |  |  |  |
| Rissoides desmaresti larvae Risso, 1816 | 0.05 | 0.00 | 0.78 |  |  |  |  |
| Lamellibrachia | 0.30 | 0.00 | 3.14 |  |  |  |  |
| *Lopadorrhynchus* sp. Grube, 1855 | 0.01 | 0.00 | 0.78 |  |  |  |  |
| Polychaeta | 0.76 | 0.00 | 3.92 |  |  |  |  |
| Polychaeta larvae | 0.13 | 0.00 | 2.35 |  |  |  |  |
| *Tomopteris septentrionalis* Steenstrup, 1849 | 0.05 | 0.00 | 1.57 |  |  |  |  |
| *Tomopteris* sp. Eschscholtz, 1825 | 0.24 | 0.00 | 1.57 |  |  |  |  |
| Chaetognatha | 6.50 | 0.00 | 23.53 |  |  |  |  |
| Anthozoa planula | 0.24 | 0.00 | 2.35 |  |  |  |  |
| *Bassia bassensis* Quoy & Gaimard, 1833 | 0.01 | 0.00 | 0.78 |  |  |  |  |
| Hydromedusae | 3.91 | 0.00 | 21.18 |  |  |  |  |
| Ephyra | 0.12 | 0.00 | 7.06 |  |  |  |  |
| Siphonophorae | 6.30 | 0.00 | 36.86 |  |  |  |  |
| Ophiuridae | 0.01 | 0.00 | 0.78 |  |  |  |  |
| Echinodermata larvae | 0.57 | 0.00 | 7.84 |  |  |  |  |
| Mollusca (Bivalvia) | 0.10 | 0.00 | 5.88 |  |  |  |  |
| Mollusca larvae | 0.15 | 0.00 | 4.71 |  |  |  |  |
| Pteropoda | 1.63 | 0.00 | 50.98 |  |  |  |  |
| *Cavolinia* spp. Abildgaard, 1791 | 0.10 | 0.00 | 1.57 |  |  |  |  |
| *Creseis* spp. Rang, 1828 | 1.07 | 0.00 | 8.63 |  |  |  |  |
| *Clio* spp. Linnaeus, 1767 | 0.16 | 0.00 | 2.35 |  |  |  |  |
| Limacinidae | 4.56 | 0.00 | 57.25 |  |  |  |  |
| Faustulidae | 0.01 | 0.00 | 0.78 |  |  |  |  |
| *Fritillaria* spp. Fol, 1872 | 4.27 | 0.00 | 30.59 |  |  |  |  |
| *Oikopleura* *dioica* Fol, 1872 | 16.70 | 0.00 | 87.06 |  |  |  |  |
| Ascidiacea larvae | 0.04 | 0.00 | 2.35 |  |  |  |  |
| Doliolidae | 2.08 | 0.00 | 17.25 |  |  |  |  |
| Salpidae | 0.27 | 0.00 | 9.41 |  |  |  |  |
| *Thalia* spp. Blumenbach, 1798 | 0.02 | 0.00 | 0.78 |  |  |  |  |
| Fish eggs | 0.79 | 0.00 | 7.06 |  |  |  |  |
| Fish larvae | 0.46 | 0.00 | 2.35 |  |  |  |  |
| Mean ΜΖ abundance (ind. m^-3^) | 381.86 |  |  |  |  |  |  |

| Table SIV. Seasonal diversity of Copepoda (number of taxa) overallat the layers 0-50m and 50-100m | | | | | | | | | | | |
| --- | --- | --- | --- | --- | --- | --- | --- | --- | --- | --- | --- |
|  |  |  |  | Winter | | Spring | | Summer | | Autumn | |
|  | Total number of taxa | 0-50m | 50-100m | 0-50m | 50-100m | 0-50m | 50-100m | 0-50m | 50-100m | 0-50m | 50-100m |
| AKR | 120 | 97 | 103 | 77 | 79 | 61 | 63 | 67 | 79 | 51 | 69 |
| PYR | 122 | 97 | 105 | 66 | 72 | 62 | 80 | 59 | 68 | 64 | 68 |
| VAS1 | 97 | 97 |  | 69 |  | 80 |  | 61 |  | 60 |  |
| VAS2 | 91 | 91 |  | 67 |  | 66 |  | 65 |  | 66 |  |

| Table SV. Seasonal variation of Shannon-Wiener (H’) and Stdev overall at the layers 0-50m and 50-100m | | | | | | | | | | | |
| --- | --- | --- | --- | --- | --- | --- | --- | --- | --- | --- | --- |
|  |  |  |  | Winter | | Spring | | Summer | | Autumn | |
|  | Mean value H’ | 0-50m | 50-100m | 0-50m | 50-100m | 0-50m | 50-100m | 0-50m | 50-100m | 0-50m | 50-100m |
| AKR | 3.02 ± 0.25 | 2.98 ± 0.28 | 3.06 ± 0.22 | 2.88 ± 0.33 | 3.01 ± 0.23 | 2.57 ± 0.51 | 3.16 ± 0.15 | 3.14 ± 0.08 | 3.10 ± 0.28 | 2.97 ± 0.29 | 2.98 ± 0.27 |
| PYR | 3.04 ± 0.21 | 3.03 ± 0.23 | 3.06 ± 0.20 | 2.84 ± 0.32 | 2.94 ± 0.29 | 3.02 ± 0.20 | 3.21 ± 0.14 | 3.23 ± 0.09 | 2.97 ± 0.11 | 3.02 ± 0.14 | 3.12 ± 0.17 |
| VAS1 | 2.94 ± 0.35 | 2.94 ± 0.35 |  | 2.88 ± 0.37 |  | 2.76 ± 0.58 |  | 3.06 ± 0.16 |  | 3.11 ± 0.13 |  |
| VAS2 | 3.05 ± 0.24 | 3.05 ± 0.24 |  | 2.89 ± 0.36 |  | 3.02 ± 0.35 |  | 3.18 ± 0.11 |  | 3.06 ± 0.13 |  |

| Table SVI. Percentage, mean relative abundance (%) and mean copepod abundance (ind. m^-3^) of the copepod orders among sampling stations. | | | | | | | |
| --- | --- | --- | --- | --- | --- | --- | --- |
| **Copepoda** | AKR_50m | AKR_100m | PYR_50m | PYR_100m | VAS1_50m | VAS2_50m | **Mean relative abundance (%)** |
| Calanoida | 71.54 | 57.26 | 71.00 | 60.67 | 77.19 | 69.84 | 68.73 |
| Cyclopoida | 28.32 | 42.60 | 29.00 | 39.24 | 22.75 | 30.14 | 31.20 |
| Harpacticoida | 0.14 | 0.11 | 0.00 | 0.08 | 0.06 | 0.02 | 0.07 |
| Monstrilloida | 0.00 | 0.00 | 0.00 | 0.01 | 0.00 | 0.00 | 0.002 |
| Siphonostomatoida | 0.00 | 0.03 | 0.00 | 0.00 | 0.00 | 0.00 | 0.004 |
| **Mean copepod abundance (ind. m^-3^)** | 314.44 | 254.97 | 272.93 | 278.01 | 383.37 | 376.86 | 313.43 |

| Table SVII. Summary of the Anova table from the multilevel pairwise comparison (~Permanova) calculated for each pair of sampling seasons. | | | | | | | |  |
| --- | --- | --- | --- | --- | --- | --- | --- | --- |
| **pairs** | **Df** | **Sums Of Sqs** | **F. Model** | **R2** | **p. value** | **p. adjusted** | **sig** | |
| Spring vs Summer | 1 | 0.222599993 | 2.754558201 | 0.115959143 | 0.004 | 0.024 | * | |
| Spring vs Autumn | 1 | 0.261197016 | 3.262377558 | 0.14654219 | 0.003 | 0.018 | * | |
| Spring vs Winter | 1 | 0.319395617 | 3.642432314 | 0.160867537 | 0.002 | 0.012 | * | |
| Summer vs Autumn | 1 | 0.188572658 | 2.935694352 | 0.127996751 | 0.001 | 0.006 | ** | |
| Summer vs Winter | 1 | 0.585089859 | 8.185674906 | 0.290419688 | 0.001 | 0.006 | ** | |
| Autumn vs Winter | 1 | 0.465958733 | 6.689957105 | 0.270958636 | 0.001 | 0.006 | ** | |

*: *p* < 0.05; **: *p* < 0.01; ***: *p* < 0.001.
